# Mammalian hydroxylation of microbiome-derived obesogen, delta-valerobetaine, to homocarnitine, a 5-carbon carnitine analog

**DOI:** 10.1016/j.jbc.2024.108074

**Published:** 2024-12-13

**Authors:** Jaclyn Weinberg, Ken H. Liu, Choon-Myung Lee, William J. Crandall, André R. Cuevas, Samuel A. Druzak, Edward T. Morgan, Zachery R. Jarrell, Eric A. Ortlund, Greg S. Martin, Grant Singer, Frederick H. Strobel, Young-Mi Go, Dean P. Jones

**Affiliations:** 1 Division of Pulmonary, Allergy, Critical Care and Sleep Medicine, Department of Medicine, Emory University School of Medicine, Atlanta, Georgia, USA; 2 Department of Biochemistry, Department of Medicine, Emory University, Atlanta, Georgia, USA; 3 Department of Pharmacology and Chemical Biology, Emory University, Atlanta, Georgia, USA; 4 Department of Chemistry, Emory University, Atlanta, Georgia, USA

**Keywords:** acyltransferase, BBOX, carnitine, δ-valerobetaine, energy metabolism, fatty acid metabolism, homocarnitine, microbiome, obesity

## Abstract

The recently discovered microbiome-generated obesogen, δ-valerobetaine (5-(trimethylammonio)pentanoate), is a 5-carbon structural analog of the carnitine precursor, γ-butyrobetaine. Here, we report that δ-valerobetaine is enzymatically hydroxylated by mammalian γ-butyrobetaine dioxygenase (BBOX) to form 3-hydroxy-5-(trimethylammonio)pentanoate, a 5-carbon analog of carnitine, which we term homocarnitine. Homocarnitine production by human liver extracts depends upon the required BBOX cofactors, 2-oxoglutarate, Fe^2+^, and ascorbate. Molecular dynamics simulations show successful docking of δ-valerobetaine and homocarnitine to BBOX, pharmacological inhibition of BBOX prevents homocarnitine production, and transfection of a liver cell line with BBOX substantially increases production. Furthermore, an *in vivo* isotope tracer study shows the conversion of ^13^C_3_-trimethyllysine to ^13^C_3_-δ-valerobetaine then ^13^C_3_-homocarnitine in mice, confirming the *in vivo* production of homocarnitine. Functional assays show that carnitine palmitoyltransferase acylates homocarnitine to acyl-homocarnitine, analogous to the reactions for the carnitine shuttle. Studies of mouse tissues and human plasma show widespread distribution of homocarnitine and fatty acyl-homocarnitines. The respective structural similarities of homocarnitine and acyl-homocarnitines to carnitine and acyl-carnitines indicate that homocarnitine could impact multiple sites of carnitine distribution and activity, potentially mediating microbiome-associated obesity and metabolic disorders.

Recent research shows that a diet-dependent mammalian obesogen, 5-(trimethylammonio)pentanoate (δ-valerobetaine), is produced from N_6_-,N_6_-,N_6_-trimethyllysine by bacteria in the mammalian intestinal microbiome (1, 2, 3). δ-Valerobetaine is a 5-carbon structural analog of γ-butyrobetaine (4), the direct biosynthetic precursor to carnitine in mammals. Carnitine is nearly ubiquitous in animal systems (4, 5) and has a fundamental role in transporting fatty acids into mitochondria for β-oxidation (6, 7, 8). δ-Valerobetaine is also widely distributed in mammalian tissues and is linked to obesity and aging in mice and humans (3, 9, 10). Importantly, previous studies show δ-valerobetaine inhibits fatty acid β-oxidation in carnitine-rich tissues including the liver, muscle, brain, and kidney (3, 11).

Mammals maintain the carnitine levels through endogenous biosynthesis, dietary intake, and renal reabsorption. Biosynthesis of carnitine from γ-butyrobetaine occurs *via* γ-butyrobetaine dioxygenase (BBOX), a 2-oxoglutarate, Fe^2+^, and ascorbate-dependent enzyme that is highly expressed in liver and kidney (12, 13). The structural similarity between δ-valerobetaine and γ-butyrobetaine (14) raises the possibility that δ-valerobetaine is similarly hydroxylated by BBOX to form a 5-carbon sructural analog of carnitine. In principle, such an analog could be acylated or transported by the same systems as those functioning for carnitine.

Here, we report the mammalian bioconversion of δ-valerobetaine to 3-hydroxy-5-(trimethylammonio)pentanoate, a structural analog one carbon longer than carnitine, which we refer to as homocarnitine (Fig. 1). To characterize this reaction, we conducted molecular dynamics simulations and combined mass spectrometry with cell-free enzyme activity assays, *in vitro* assessment using a BBOX inhibitor, a BBOX overexpressing liver cell line, and isotope tracer analyses in mice. Enzyme activity, isotope tracer, and mass spectrometry studies confirm the generation of palmitoyl- and acetyl-homocarnitine in reactions analogous to that for the generation of acyl-carnitines. Finally, we detect homocarnitine and acetyl-homocarnitine in human samples and show a correlation with δ-valerobetaine.

**Figure 1:**
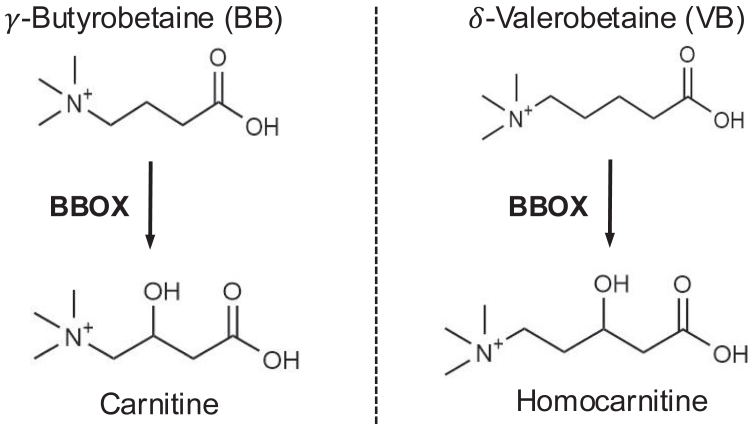
**Scheme showing structures of γ-butyrobetaine and its BBOX-generated product, carnitine, and δ-valerobetaine with its analogous BBOX-generated product, homocarnitine**.

## Results

### Molecular dynamics simulations of BBOX1 interactions with δ-valerobetaine and homocarnitine

**Figure 2:**
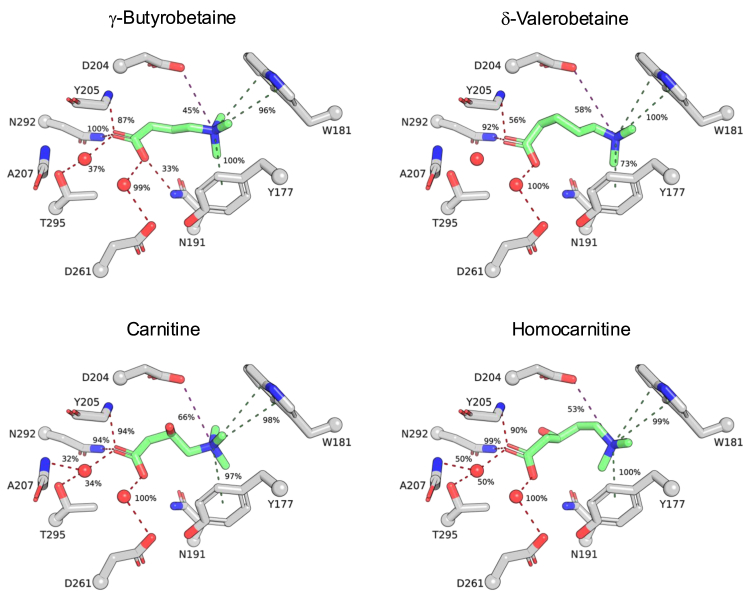
**Molecular dynamics simulations of BBOX1 interactions with δ-valerobetaine and homocarnitine**. Modeling studies conducted with Maestro and Glide to dock γ-butyrobetaine and its BBOX-generated product, carnitine, and δ-valerobetaine and its proposed BBOX-generated product, homocarnitine in the catalytic site of BBOX1 (PDB: 3O2G (17)). Residues with > 30% interaction times across three simulations are shown. Interaction times between molecule atoms and BBOX residues are represented as the average percentage of three simulations that contacts are made. All key residues with interaction fraction times and indicated chemical bond type are available in Fig. S2. *Green* = carbon atoms on substrate or product. *Red atoms* = oxygen. *Blue atoms* = nitrogen. *Red spheres* = water. *Red dashed lines* = hydrogen bond. *Purple dashed lines* = ionic interaction. *Green dashed lines* = cation-π interaction.

We conducted a similarity ensemble approach (SEA) to obtain an unbiased determination of proteins likely to interact with δ-valerobetaine. SEA compares an input structure with an online database of known ligand-protein pairs to determine potential protein hits based on structural similarity (15). Results showed δ-valerobetaine, a 5-carbon analog of γ-butyrobetaine, could interact with multiple proteins, with the highest likelihood for interaction with γ-butyrobetaine dioxygenase, BBOX1 (Fig. S1). To visualize the positioning of δ-valerobetaine compared to γ-butyrobetaine in the active site of BBOX, all-atom molecular dynamics simulations were conducted (summarized in Fig. 2; full analysis in Fig. S2). The trimethylammonio group of γ-butyrobetaine and δ-valerobetaine made cation-π interactions with W181 and Y177 and an ionic interaction with D204, while the carboxylates hydrogen bond with other residues directly or through waters. Previous studies show the carboxyl group is a required component of the BBOX substrate structure, but the positioning of the trimethylammonio group is not as impactful on binding and catalysis (16, 17). Mean contact times with Y205, Y177, N191, and T295 were reduced for δ-valerobetaine compared to γ-butyrobetaine, consistent with differences in micromolar affinity to BBOX1 (2). The addition of hydroxyl to δ-valerobetaine at the β carbon resulted in the orientation of hydroxyl toward active site Zn^2+^, analogous to the hydroxyl position of carnitine.

### Metabolism of δ-valerobetaine to homocarnitine

**Figure 3:**
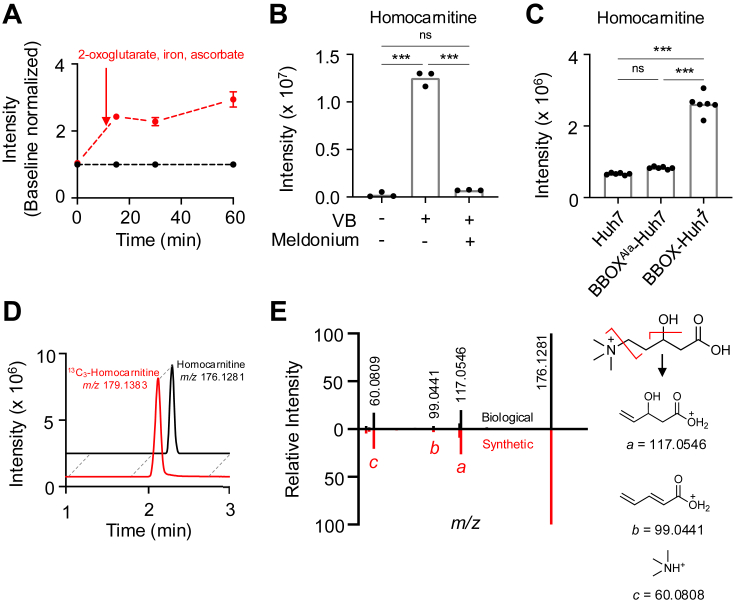
**Bioconversion of δ-valerobetaine to homocarnitine**. *A*, intensity of *m/z* 176.1281 corresponding to homocarnitine in mouse liver S9 fractions incubated with 50 μM δ-valerobetaine and either vehicle (*black*) or BBOX cofactors (*red*) (N = 3). BBOX cofactors consisted of 3 mM 2-oxoglutarate, 0.25 mM ferrous chloride, and 10 mM sodium ascorbate. Means and standard error of the mean (SEM) are shown. *B*, homocarnitine production (*m/z* 176.1281) in primary rat hepatocytes incubated for 6 h with vehicle or 100 μM δ-valerobetaine with or without 100 μM meldonium, a competative BBOX inhibitor. *C*, homocarnitine (*m/z* 176.1281) production by Huh7 and stably transfected BBOX^Ala^-Huh7 and BBOX-Huh7 cell lines incubated for 4 h with 100 μM δ-valerobetaine. *D*, extracted ion chromatogram from HILIC/ESI+ analysis of BBOX-Huh7 cell lysate shows coelution of homocarnitine (*m/z* 176.1281) (*black*) with synthetic 10 μM ^13^C_3_-homocarnitine (*m/z* 179.1383) (*red*). *E*, mirror plot showing MS^2^ ion dissociation spectra of homocarnitine (HCD 35%) from BBOX-Huh7 cell lysate (*black; top*) and synthetic homocarnitine at 10 μM in saline extracted by acetonitrile (*red; bottom*). On the *right*, proposed structures and exact masses (Da) of observed product ions (*A*: 117.0546, *B* = 99.0441, *C* = 60.0809) resulting from 2 fragmentation sites on homocarnitine’s structure (*red lines*) are presented; note that *m/z* 99.0441 could also form a cyclic structure. Group differences in panels *B* and *C* were determined with a one-way ANOVA followed by Tukey’s test of multiple comparisons (*p* ≤ 0.001 = ∗∗∗, ns = *p* > 0.05).

To test for the conversion of δ-valerobetaine (*m/z* 160.1332, M^+^) to homocarnitine (*m/z* 176.1287, M^+^), we used a mouse liver S9 preparation (18). S9 fractions consist of the enzyme-rich cytosolic and microsomal portions of the cell and rely on the supplementation of cofactors for enzyme activation (19). After liver S9 fractions were pre-incubated with δ-valerobetaine, mass spectrometry with positive electrospray ionization (ESI+) showed that with cofactors added, an intensity at *m/z* 176.1281 (Δ 3.4 ppm) increased with time (Fig. 3*A*), consistent with hydroxylation of δ-valerobetaine to homocarnitine (M^+^ with elemental composition C_8_H_18_NO_3_^+^), while no increase in signal intensity was observed over time at *m/z* 176.1287 in the absence of added cofactors (2-oxoglutarate, Fe^2+^, ascorbate) for BBOX (Fig. 3*A*).

To test for BBOX conversion of δ-valerobetaine to homocarnitine in cell culture, we treated primary rat hepatocytes with vehicle or δ-valerobetaine with or without meldonium, a competitive BBOX inhibitor (IC_50_ = 60 μM) (16). Mass spectrometry analysis after a 6 h incubation showed an increase in homocarnitine (*m/z* 176.1281) that was prevented by meldonium cotreatment (Fig. 3*B*). Additional confirmation was provided by a human hepatoma Huh7 cell line (BBOX-Huh7) expressing V5 epitope-tagged BBOX1. Cells were characterized by Western blotting to confirm expression of BBOX-V5 (Fig. S3), and BBOX activity was confirmed by measuring carnitine production from a 4 h treatment of γ-butyrobetaine (Fig. S4). Incubation of BBOX-Huh7 cells with δ-valerobetaine showed increased homocarnitine production compared to non-transfected Huh7 cells (Fig. 3*C*). Incubation of δ-valerobetaine in Huh7 cells with catalytically inactive BBOX due to mutation of active site Asp204 to Ala204 (17) showed no increased production of homocarnitine (Fig. 3*C*).

With hydrophilic interaction chromatography (HILIC), homocarnitine (*m/z* 176.1281) from BBOX-Huh7 cell lysate coeluted with synthetic ^13^C_3_-homocarnitine (*m/z* 179.1383) (Fig. 3*D*). Additional validation of homocarnitine generation was performed using MS^2^ ion dissociation mass spectrometry of *m/z* 176.1281, and the result showed product ions at *m/z* 60.0809 (C_3_H_10_N^+^, Δ 1.6 ppm), *m/z* 99.0441 (C_5_H_7_O_2_^+^, Δ 0 ppm), and *m/z* 117.0546 (C_5_H_9_O_3_^+^, Δ 0 ppm), consistent with predicted product ions of homocarnitine (Fig. 3*E*). This ion dissociation pattern of homocarnitine is analogous to the dissociation pattern of carnitine [*m/z* 60.0809, *m/z* 85.0284, *m/z* 103.0389 (20)] with the addition of -CH_2_ for the two larger product ions (*m/z* 99.0441, *m/z* 117.0546).

### *In vivo* production of homocarnitine

**Figure 4:**
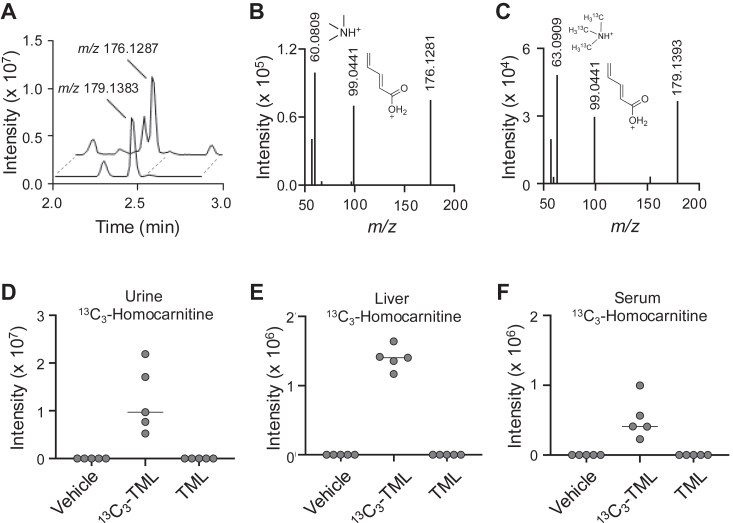
**Isotope tracer study of homocarnitine formation in mice.***A*, extracted ion chromatogram from HILIC/ESI+ for homocarnitine (*m/z* 176.1281) in urine of mice gavaged with 100 mg/kg trimethyllysine (TML) coelutes with ^13^C_3_-homocarnitine (*m/z* 179.1383) in urine of mice gavaged with 25 mg/kg ^13^C_3_-trimethyllysine (^13^C_3_-TML). *B*, MS^2^ ion dissociation (HCD 25%) spectrum of *m/z* 176.1281 in mouse urine shows *m/z* 60.0809 and *m/z* 99.0441 product ions from homocarnitine. *C*, MS^2^ ion dissociation (HCD 25%) spectrum of *m/z* 179.1383 for ^13^C_3_-homocarnitine in urine of mice given ^13^C_3_-TML shows corresponding product ion *m/z* 63.0909 representing the labelled carbons and other homocarnitine product ion at *m/z* 99.0441. Relative tissue abundances of labelled ^13^C_3_-homocarnitine (*m/z* 179.1383) from the tracer study are provided for (*D*) urine, (*E*) liver, and (*F*) serum.

To test for *in vivo* production, we conducted an isotope tracer study in mice with non-labeled trimethyllysine (TML) and ^13^C_3_-trimethyllysine (^13^C_3_-TML), a precursor previously shown to be converted *in vivo* to δ-valerobetaine (3, 21). Mass spectral analysis of mouse urine showed production of ^13^C_3_-homocarnitine (*m/z* 179.1383) in ^13^C_3_-TML treated mice with a 3.0102 mass shift (Δ1.7 ppm) relative to homocarnitine (*m/z* 176.1281) produced in TML-treated mice (Fig. 4*A*). Ion dissociation (MS^2^) spectra further showed the expected mass shift of 3.0099 for the carbon-labeled quaternary amine of homocarnitine (Fig. 4, *B* and *C*). The other detected product ion consistent with homocarnitine was *m/z* 99.0441 (Fig. 4, *B* and *C*). The ^13^C_3_-labeled form of homocarnitine was detected in the urine, liver and serum of mice only when treated with ^13^C_3_-TML (Fig. 4, *D*–*F*).

**Figure 5:**
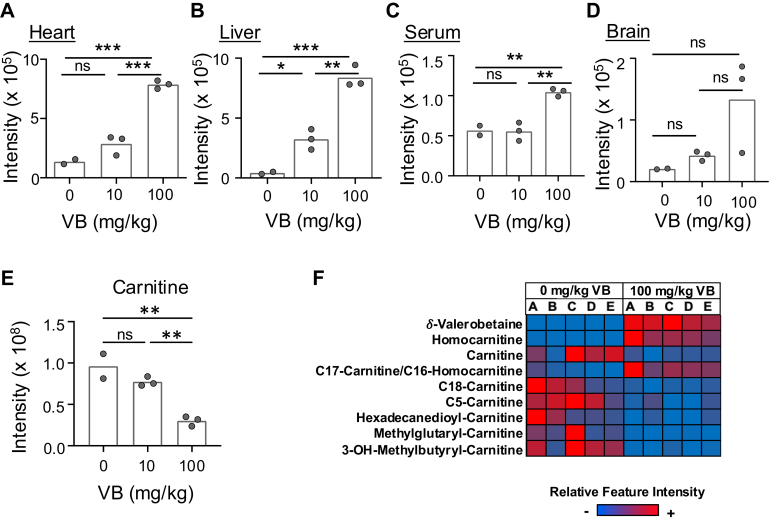
**Effectof δ-valerobetaine on tissue levels of homocarnitine and serum carnitine metabolites.** The effect of oral δ-valerobetaine dose on relative abundance of homocarnitine in mouse heart (*A*), liver (*B*), serum (*C*), and brain (*D*) with means shown. *E*, serum levels of carnitine (*m/z* 162.1125) at each dose of δ-valerobetaine. *F*, relative intensities of spectral features are indicated from low (*blue*) to high (*red*) across 5 mouse replicates treated with saline or 100 mg/kg δ-valerobetaine. Relative intensities were calculated individually for each metabolite in each row. Female data are presented; male mice showed similar response (data not shown). Group differences in panels (*A*–*E*) were determined with a one-way ANOVA followed by Tukey’s test of multiple comparisons (*p* ≤ 0.05 = ∗, *p* ≤ 0.01 = ∗∗, *p* ≤ 0.001 = ∗∗∗, ns = *p* > 0.05).

To test for tissue distribution of homocarnitine, a dose-response mouse study including a supraphysiological concentration of δ-valerobetaine showed increased homocarnitine (*m/z* 176.1281) in heart (Fig. 5*A*), liver (Fig. 5*B*), and serum (Fig. 5*C*); a similar but non-significant pattern was observed in brain (Fig. 5*D*). Quantification showed that serum concentration of δ-valerobetaine was between 8 and 14 μM for control mice and 224 and 379 μM for the 100 mg/kg δ-valerobetaine-treated mice. Serum homocarnitine concentration was 3 μM for control mice and 4 to 6 μM for 100 mg/kg δ-valerobetaine-treated mice. A significant decrease in carnitine was observed in mice treated with 100 mg/kg δ-valerobetaine (Fig. 5*E*), consistent with previous findings (3). The 100 mg/kg δ-valerobetaine dose also induced a decrease in acyl-carnitines as previously shown (2, 3, 22) (Fig. 5*F*). One of the mass spectral features (*m/z* 414.3567), annotated as C_17_-acyl-carnitine, differed from others by increasing in response to δ-valerobetaine (Fig. 5*F*). Because C_17_-acyl-carnitine is isomeric with a possible C_16_-acylation product of homocarnitine, *i.e.*, palmitoyl-homocarnitine, this observation suggested that homocarnitine can be acylated to form fatty acyl-homocarnitines. Further, the δ-valerobetaine-induced decrease in carnitine and increase in C_17_-carnitine/C_16_-acyl-homocarnitine (*m/z* 414.3567) suggested that this feature might be primarily C_17_-carnitine in the control mice but C_16_-acyl-homocarnitine in the treated mice.

### Acylation of homocarnitine

**Figure 6:**
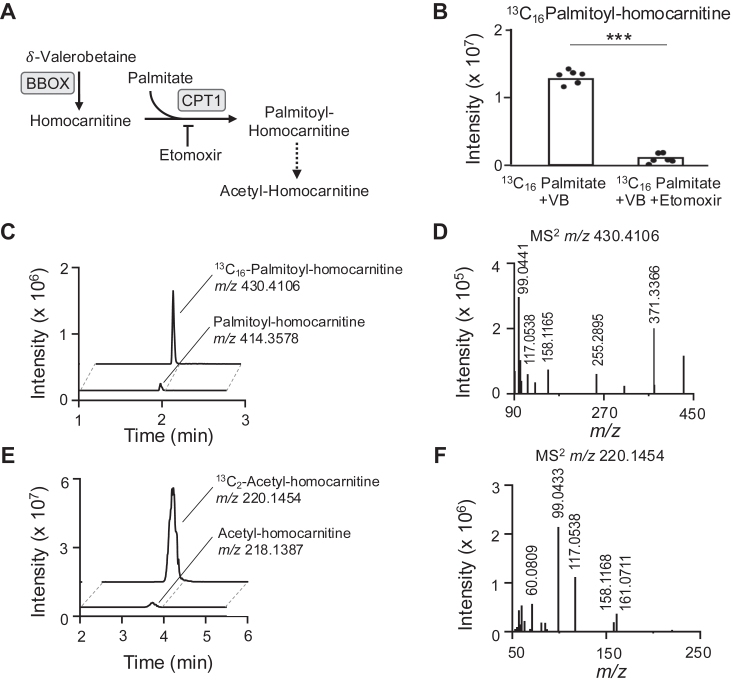
**Formation and detection of fatty acyl-homocarnitines.***A*, scheme showing CPT1-catalyzed fatty acylation of homocarnitine produced from δ-valerobetaine by BBOX and CPT1 inhibition by etomoxir. *B*, levels of ^13^C_16_-palmitoyl-homocarnitine (*m/z* 430.4106) in BBOX-Huh7 cells preincubated with 200 μM δ-valerobetaine then treated with 40 μM [^13^C_16_]palmitate plus vehicle or 40 μM etomoxir (*p* < 0.001). *C*, extracted ion chromatogram from HILIC/ESI+ shows coelution of palmitoyl-homocarnitine (*m/z* 414.3578) and ^13^C_16_-palmitoyl-homocarnitine (*m/z* 430.4106) in BBOX-Huh7 cells treated with δ-valerobetaine and [^13^C_16_]palmitate. *D*, MS^2^ ion dissociation of ^13^C_16_-palmitoyl-homocarnitine (*m/z* 430.4106) in BBOX-Huh7 cells treated with δ-valerobetaine and [^13^C_16_]palmitate shows expected product ions. Proposed structures for observed ^13^C_16_-palmitoyl-homocarnitine product ions are described in Fig. S6. *E*, extracted ion chromatogram of ^13^C_2_-acetyl-homocarnitine (*m/z* 220.1454) and acetyl-homocarnitine (*m/z* 218.1387) in BBOX-Huh7 cells treated with δ-valerobetaine and [^13^C_16_]palmitate. *F*, MS^2^ ion dissociation of ^13^C_2_-acetyl-homocarnitine (*m/z* 220.1454) in BBOX-Huh7 cells treated with δ-valerobetaine and [^13^C_16_]palmitate shows expected product ions. Proposed structures for observed ^13^C_2_-acetyl-homocarnitine product ions are described in Fig. S7. Chromatograms and MS^2^ spectra for *in vivo* heart palmitoyl-homocarnitine (*m/z* 414.3578) and acetyl-homocarnitine (*m/z* 218.1387) are provided in Fig. S9; proposed structures for respective product ions are provided in Figs. S6 and S10. Results from a similar experiment conducted in primary rat hepatocytes with pharmacological CPT1 and BBOX inhibition is available in Fig. S8.

We examined structural similarity for protein binding using SEA (15) and found that homocarnitine is likely to interact with carnitine palmitoyl-transferase 1 (CPT1) (Fig. S5), which performs the rate-limiting step in oxidation of long-chain fatty acids (6). To test whether homocarnitine undergoes acylation by CPT1A similarly to carnitine (Fig. 6*A*) (6), BBOX-Huh7 cells were pre-incubated overnight with δ-valerobetaine to allow formation of homocarnitine and then treated with [^13^C_16_]palmitate for 2 h without or with the CPT1 inhibitor, etomoxir (Fig. 6*A*). Results show production of ^13^C_16_-palmitoyl-homocarnitine (*m/z* 430.4106; ^13^C_16_^12^C_8_H_48_NO_4_^+^, Δ 0 ppm) in a reaction that was inhibited by etomoxir (*p* < 0.001) (Fig. 6*B*). Chromatography showed coelution of ^13^C_16_-palmitoyl-homocarnitine (*m/z* 430.4106, peak area = 1.97E7) and ^12^C_16_-palmitoyl-homocarnitine (*m/z* 414.3578, peak area = 2.85E6) (Fig. 6*C*). Ion dissociation (MS^2^) spectra for ^13^C_16_-palmitoyl-homocarnitine (*m/z* 430.4106) showed an accurate mass *m/z* matching the predicted acylated homocarnitine product ion (*m/z* 158.1177, C_8_H_16_NO_2_^+^) along with other expected product ions for ^13^C_16_-palmitoyl (*m/z* 371.3366) and free (*m/z* 99.0441 and 117.0538) homocarnitine (Figs. 6*D* and S6).

Results from the incubation of BBOX-Huh7 cells with δ-valerobetaine and [^13^C_16_] palmitate also showed an accurate mass match to ^13^C_2_-acetyl-homocarnitine (*m/z* 220.1454, peak area = 9.59E8) which co-eluted with ^12^C_2_-acetyl-homocarnitine (*m/z* 218.1387, peak area = 1.08E7) (Fig. 6*E*). MS^2^ spectra for *m/z* 220.1454 (^13^C_2_^12^C_7_H_20_NO_4_^+^, Δ 0 ppm) included product ions *m/z* 161.0711 and 158.1168 consistent with being derived from ^13^C_2_-acetyl-homocarnitine and other product ions characteristic of homocarnitine (*m/z* 60.0809, 99.0433, 117.0538) (Figs. 6*F* and S7). Analysis in primary rat hepatocytes showed that palmitoyl-homocarnitine (*m/z* 414.3578) generation increased with the addition of δ-valerobetaine and was inhibited by the CPT1 inhibitor, etomoxir, and BBOX inhibitor, meldonium (Fig. S8*A*). Acetyl-homocarnitine (*m/z* 218.1387) increased with δ-valerobetaine treatment, and this increase was also inhibited by CPT1 and BBOX inhibitors (Fig. S8*B*).

### Detection of acyl-homocarnitines *in vivo*

To determine whether acyl-homocarnitine species are formed *in vivo*, heart tissue from mice treated with 100 mg/kg δ-valerobetaine were analyzed for detection of palmitoyl-homocarnitine (*m/z* 414.3578) and acetyl-homocarnitine (*m/z* 218.1387). Results showed a signal at *m/z* 414.3578 coeluted on HILIC with palmitoyl-carnitine (*m/z* 400.3421) (Fig. S9*A*), and the respective MS^2^ spectrum of *m/z* 414.3578 showed acylated homocarnitine product ions (*m/z* 158.1177 and 355.2846) as well as a free homocarnitine product ion (*m/z* 99.0442) (Figs. S6 and S9*B*). Acetyl-homocarnitine (*m/z* 218.1387) eluted slightly earlier than acetyl-carnitine (*m/z* 204.1230) (Fig. S9*C*), and the respective MS^2^ spectrum for acetyl-homocarnitine (*m/z* 218.1387) showed a product ion from acylated homocarnitine (*m/z* 158.1177) and others from homocarnitine (*m/z* 60.0810, 99.0441, 117.0547) (Figs. S7 and S9*D*). Two product ions (*m/z* 85.0284 and 159.0653) were observed which could be derived from even-chained acyl-homocarnitines or isomeric odd-chained acyl-carnitines (Fig. S10). Together, these data show that in addition to formation of homocarnitine from the microbial product δ-valerobetaine, cells and mice have capabilities to acylate homocarnitine to palmitoyl-homocarnitine and acetyl-homocarnitine.

### Homocarnitine and acyl-homocarnitine in humans

**Figure 7:**
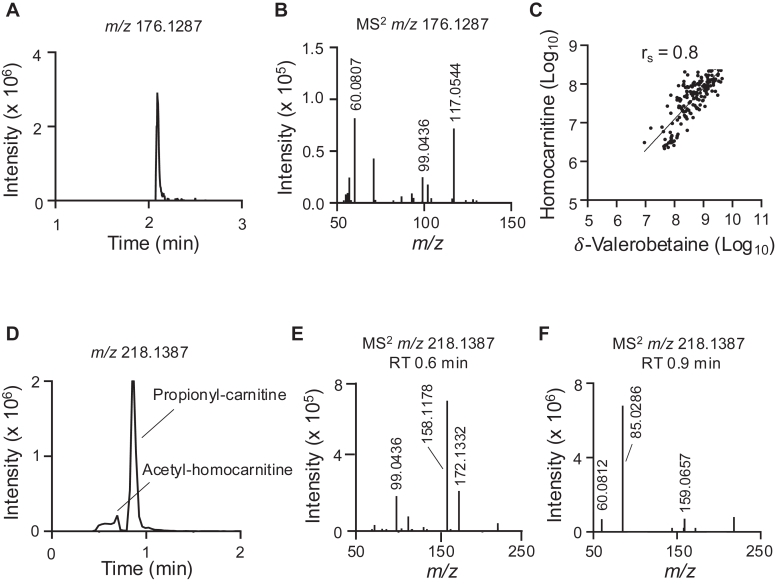
**Detection of acetyl-homocarnitine in human samples.***A*, extracted ion chromatogram of homocarnitine in a pooled reference sample of human plasma. *B*, MS^2^ ion dissociation of homocarnitine in a pooled reference sample of human plasma shows product ions characteristic of homocarnitine (see Fig. 3*E*). *C*, Spearman correlation (r_s_ = 0.8, *p* < 0.001) of δ-valerobetaine and homocarnitine in human urine (N = 165). *D*, extracted ion chromatogram of the isomeric species acetyl-homocarnitine and propionyl-carnitine (*m/z* 218.1387) in a pooled reference sample of human plasma. *E*, MS^2^ ion dissociation spectrum of the first peak representing acetyl-homocarnitine (*m/z* 218.1387; retention time (RT) = 0.6 min) with expected product ions (*m/z* 99.0436, 158.1178, 172.1332) in a pooled reference sample of human plasma. *F*, MS^2^ ion dissociation spectrum of the second peak representing propionyl-carnitine (*m/z* 218.1387; RT = 0.9 min) with expected product ions (*m/z* 60.0812, 85.0286, 159.0657) in a pooled reference sample of human plasma. The intensity of product ion *m/z* 144.1022 for propionyl-carnitine is also detectable but low in this spectrum. Relative intensity of this product ion was higher in other samples. Estimated concentrations of homocarnitine in human plasma and urine are provided in Fig. S11. Correlation of plasma δ-valerobetaine with homocarnitine is provided in Fig. S12. Proposed structures of acetyl-homocarnitine and propionyl-carnitine product ions are available in Fig. S10.

To determine if homocarnitine and acyl-homocarnitines are present in humans, plasma and urine samples were analyzed. In pooled human reference plasma (23), a signal for homocarnitine (*m/z* 176.1281) had the same retention time and MS^2^ ion dissociation spectrum as the synthetic standard (compare Fig. 7*A* to Fig 3*D*; and Fig. 7*B* to Fig. 3*E*). The concentration of homocarnitine in human plasma was 0.00 to 1.66 μM with a mean concentration of 0.15 μM and a standard deviation of 0.33 μM (Fig. S11*A*). The concentration of homocarnitine was higher in human urine at 0.05 to 7.36 μM with a mean concentration of 1.11 μM and standard deviation of 1.31 μM (Fig. S11*B*). Homocarnitine correlated strongly to δ-valerobetaine in urine (Spearman r_s_ = 0.8, *p* < 0.001; Fig. 7*C*) but had only a small correlation in plasma (Fig. S12).

Acetyl-homocarnitine (*m/z* 218.1387) was also observed in human plasma, with a slightly earlier retention time on HILIC than its isomer, propionyl-carnitine (*m/z* 218.1387) (Fig. 7*D*). Importantly, the relative intensities of these precursors indicate that acetyl-homocarnitine is likely much less abundant than propionyl-carnitine (Fig. 7*D*). Representative ion dissociation (MS^2^) spectra of *m/z* 218.1387 showed that the earlier peak had characteristic *m/z* 99.0436 and 158.1778 product ions of acetyl-homocarnitine (Figs. 7*E* and S10) while the later peak had characteristic *m/z* 60.0812, 85.0286, 159.0657 product ions of propionyl-carnitine (Figs. 7*F* and S10). Another prominent product ion, *m/z* 172.1332, consistent with acetyl-homocarnitine and propionyl-carnitine’s structures (Fig. S10), was detected in human plasma matrices.

## Discussion

**Figure 8:**
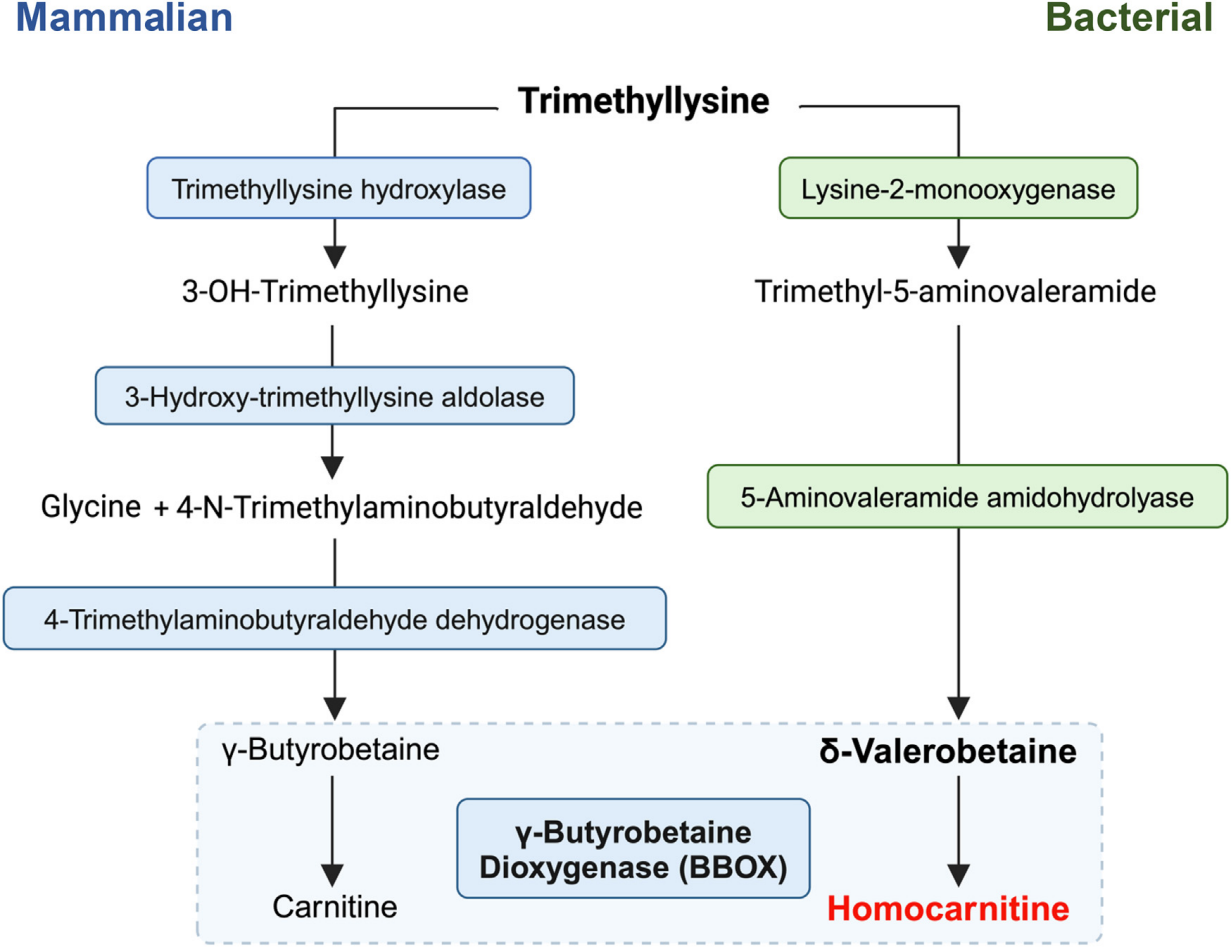
**Proposed metabolic pathway of homocarnitine synthesis.** (*Left*) Prior research shows that mammalian enzymes produce γ-butyrobetaine from trimethyllysine (71, 72) through a sequence of hydroxylation (73, 74), cleavage (75), and dehydrogenation (76) reactions. (*Right*) Recent studies identified two bacterial enzymes in the intestinal microbiome, lysine 2-monooxygenase (DavB; EC 1.13.12.2) and 5-aminovaleramide amidohydrolase (DavA; EC 3.5.1.30), that metabolize trimethyllysine to δ-valerobetaine (2, 21, 22). It was previously established that these two enzymes metabolize lysine to 5-aminovaleramide then to 5-aminovalerate. Similarly, lysine 2-monooxygenase converts trimethyllysine to trimethyl-5-aminovaleramide which is then converted to δ-valerobetaine by 5-aminovaleramide amidohydrolase (2, 22, 77, 78, 79). The present research shows that δ-valerobetaine is hydroxylated to form homocarnitine in a subsequent step catalyzed by mammalian γ−butyrobetaine dioxygenase (BBOX) (12, 80, 81), the enzyme that converts γ−butyrobetaine to carnitine. Enzymes are listed in *blue* (mammalian) and *green* (bacterial) boxes. Created with BioRender.com.

The present study demonstrates that the microbiome-derived obesogen, δ-valerobetaine, undergoes mammalian hydroxylation by BBOX to form homocarnitine, a structural analog of carnitine. Widespread tissue distribution and conversion of homocarnitine to acyl-homocarnitines defines a new metabolic pathway that parallels the pathway for carnitine (Fig. 8). In this pathway, dietary intake of lysine and trimethyllysine (TML) (1) supports microbiome generation of δ-valerobetaine (2, 3, 22) and subsequent mammalian host production of homocarnitine (Figure 3, Figure 4, Figure 5, Figure 6, Figure 7) and fatty acyl-homocarnitines (Figs. 6 and 7). Comparison of the carnitine and homocarnitine pathways suggests that the homocarnitine pathway could be a relic of an ancestral holobiome (24) fatty acid oxidation system that is undergoing replacement by a more efficient γ-butyrobetaine-carnitine system (3, 25). Alternatively, the pathway could function along with δ-valerobetaine to balance bioenergetics of the holobiome in the short term by regulating mitochondrial β-oxidation and in the long term by storage of fatty acids in depot fat (26, 27).

Additional functional studies will be needed to understand the relationship of the homocarnitine pathway to the activities of δ-valerobetaine. δ-Valerobetaine is a diet-dependent obesogen (3) associated with metabolic dysregulation in humans, including cardiovascular disease (2, 28), nonalcoholic fatty liver disease (3, 22), diabetes (29), and obesity (3). The mechanism(s) by which δ-valerobetaine drives metabolic disorders include decreasing systemic carnitine and γ-butyrobetaine, through occupation of a shared organic cation transporter SLC22A5 (OCTN2) for renal re-uptake (3, 11, 22, 30) and/or competitive binding to BBOX (22) resulting in decreased carnitine production (3, 22). Exogenous supplementation with carnitine has been shown to reverse this δ-valerobetaine-induced carnitine decrease and negative impact on fatty acid oxidation and fatty liver (3). Carnitine supplementation to manage obesity and cardiovascular disease or enhance physical performance has shown mixed results (31, 32, 33, 34), which could involve variable effects of δ-valerobetaine or homocarnitine on carnitine transport, synthesis, and acylation.

The activity of BBOX is determined by the availability of substrates (35, 36, 37), potassium ions (38) reducing agents [catalase (39), ascorbate (40)] for active site Fe^2+^ (17), and regulation of gene expression (41, 42). Molecular dynamics simulations for substrate interactions show less contact time for δ-valerobetaine (Figs. 2 and S2), which is consistent with a lower affinity of δ-valerobetaine compared to γ-butyrobetaine for BBOX (16). Experimental studies with a γ-butyrobetaine and δ-valerobetaine mixture showed predominantly carnitine production (Fig. S13) until an apparent threshold for δ-valerobetaine concentration was achieved for homocarnitine production (Fig. S14). Human concentrations of δ-valerobetaine are 3 to 22 μM in the human heart (11), 1 μM in a pooled human plasma (43), and about 5 μM in human milk (21). Concentrations of γ-butyrobetaine are generally higher than δ-valerobetaine in humans, with ratio dependent upon age (9, 44), diet (3, 11, 45), and microbiome status (3, 9). In ruminants, however, estimates for δ-valerobetaine in muscle tissue (500 μmoles/kg) and milk (60 μmoles/kg) are much higher than human levels (21, 46), indicating that relative rates of homocarnitine generation are greater in ruminants. Production rates of carnitine, homocarnitine, and acylated derivatives depend upon the expression of several genes integral to fatty acid oxidation including BBOX (41, 42) and carnitine acyltransferases (35), which is controlled by peroxisome proliferation-activated receptor α (PPARα). PPARα is activated by free fatty acids (47) and by δ-valerobetaine (2, 22).

**Figure 9:**
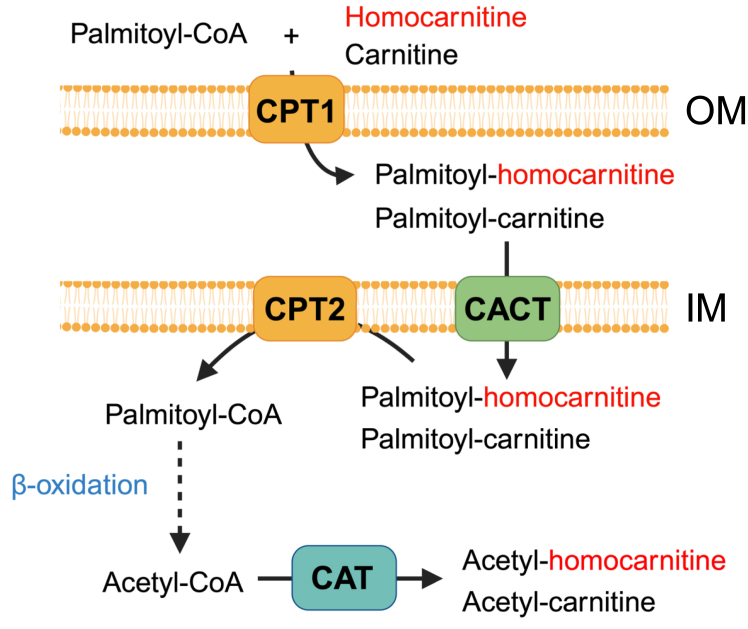
**Proposed metabolic pathway of homocarnitine acylation.** The present research shows that homocarnitine is acylated to form palmitoyl-homocarnitine, with pharmacologic evidence that this is catalyzed by carnitine palmitoyl transferase-1 (CPT1). CPT1 is an enzyme that exists in different tissue-specific forms and catalyzes fatty acyl-carnitine formation from acyl-CoA’s and carnitine (4, 82). Carnitine-acylcarnitine translocase (CACT) (83, 84) transports free and acylated-carnitines into the mitochondrial matrix where CPT2 (50) replaces the carnitine on a fatty acyl chain with a CoA to prepare for β-oxidation. Similarly, the present research shows existence of acetyl-homocarnitine likely indicating that homocarnitine and/or palmitoyl-homocarnitine can enter the mitochondria *via* CACT. The final product of β-oxidation is acetyl-CoA which can be converted to acetyl-carnitine or acetyl-homocarnitine by carnitine acetyltransferase (CAT or CrAT) (52). Created with BioRender.com. OM, outer mitochondrial membrane; IM,: inner mitochondrial membrane.

The discovery of acylated homocarnitines suggests that homocarnitine could compete with carnitine at the carnitine palmitoyltransferases CPT1A (liver), CPT1B (skeletal and cardiac muscle) (48), and CPT1C (brain) (49), interfere with mitochondrial carnitine-acylcarnitine translocase (CACT), or impair removal of carnitine from acyl-carnitines in the inner mitochondria membrane by CPT2 (50) (Fig. 9). The presence of short-chain acetyl-homocarnitine, likely generated by carnitine acetyltransferase (CAT), suggests that functional interactions could occur with free fatty acid, fatty acyl-CoA, and fatty acyl-carnitine homeostasis inside and outside of the mitochondria (51). The inability of etomoxir to completely suppress acetyl-homocarnitine formation (Fig. S8), unlike palmitoyl-homocarnitine formation, suggests that free homocarnitine enters the mitochondria and/or peroxisome to undergo acylation by carnitine acetyltransferase (CAT) instead of entering only by palmitoyl-homocarnitine (52). Together, the existence of these species suggests homocarnitine plays a role in long-chain fatty acyl shuttling across the outer mitochondrial membrane and overall cellular fatty acid management.

Odd-chained fatty acyl-carnitines with the same molecular mass as even-chained fatty acyl homocarnitines are widespread in humans. While odd-chained acyl carnitines exist at lower concentrations than even-chained acyl carnitines, they result from both endogenous biosynthesis and dietary consumption, especially from ruminant-based sources (21, 53, 54, 55). Endogenous biosynthesis of odd-chained acyl-carnitines, most commonly C_15_ and C_17_ derivatives, are synthesized with propionyl-CoA as the starting material instead of acetyl-CoA, which is used for even chains (53, 54, 56). Measurement of odd-chained acyl-carnitines by MS^2^ based upon the product ions *m/z* 60.0808, 85.0284, 159.0652, or 172.1332, miss detection of acylated homocarnitine in cell, mouse, or human matrices (Fig. S10). In contrast, MS^2^ analyses based upon the acyl-homocarnitine product ion (*m/z* 158.1178) effectively distinguish fatty acyl-homocarnitines from the isomeric odd-chained acyl-carnitines (carnitine product ion, *m/z* 144.1022) (Fig. S10). Absolute quantification, pharmacokinetics, and improved understanding of the breadth of isomeric species (57, 58) will be valuable in translating laboratory studies to useful clinical laboratory tests using microbiome-derived metabolite panels for microbiome and carnitine interventions (59, 60, 61).

In summary, the current study establishes that the intestinal microbial product δ-valerobetaine is biotransformed by mammals into homocarnitine, a structural analog of carnitine. This metabolite is converted to fatty acyl-homocarnitines by carnitine palmitoyl transferase, establishing a new metabolic pathway analogous to the carnitine pathway. Most critically, structural similarity of homocarnitine and acyl-homocarnitines to carnitine and acyl-carnitines indicates that homocarnitine could impact multiple sites of carnitine distribution and activity.

## Experimental procedures

### Human participants

Existing EDTA plasma and urine samples from the Center for Health Discovery and Well-Being (CHDWB) cohort were used for this research. Samples were collected for research purposes with informed consent under a study protocol (IRB00007243) abiding by the Declaration of Helsinki principles and reviewed and approved by the Emory University Investigational Review Board. All samples were fully de-identified. Adults were recruited from the Emory University community, including diverse races, ethnicities, and genders, and were self-described as healthy. Participants were excluded if they had a poorly treated chronic disease or an acute illness within 12 weeks of the study data collection, were pregnant or breastfeeding, or had a cancer diagnosis within 5 years. Samples were collected between 2009 and 2014 and stored at −80 °C until processing for analysis. The subset selected to test for the presence of homocarnitine was random. For metabolite extraction, 70 μl of urine or plasma was combined with 140 μl ice-cold HPLC-grade acetonitrile (Sigma-Aldrich, 900667) containing 2.5% of an isotopically labeled standard mix (23) and then vortexed. Samples were incubated on ice for 30 min before centrifugation at 14,000*g* for 10 min at 4 °C. Supernatants were transferred to autosampler vials and placed in an autosampler at 4 °C for liquid chromatography-high resolution mass spectrometry. For sample preparation for additional acquisition of MS^2^ spectra of acetyl-homocarnitine in human plasma, an alternative extraction routine was used to remove lipids and increase analyte concentration. Pooled plasma (15 ml) from Innovative Research Inc (Fisher Scientific, 50-203-6374) was frozen and then lyophilized. The lyophilized plasma was packed between cotton in a Soxhlet extraction apparatus attached to a cold-water condenser. A flask heated to 65 °C below the Soxhlet apparatus was filled with 150 ml HPLC-grade chloroform (Sigma-Aldrich, 650498) for lipid removal and run for 8 h followed by 8 h of 150 ml HPLC-grade methanol (Sigma-Aldrich, 34860). Aliquots of the methanol extraction phase were transferred to autosampler vials and maintained at 4 °C for mass spectrometry. Analysis was completed with a 10 μl injection volume.

### Animals

Animal experiments were performed under Emory University IACUC animal protocol ID 201700320 and complied with all relevant regulations regarding the use of research animals. C57BL/6J male and female mice (Jackson Laboratory, 000664) were housed in clean facilities, fed standard mouse diet (LabDiet, Laboratory Rodent Diet 5001) ad libitum. Mice were injected intraperitoneally with PBS with or without additions as indicated. In a high-dose δ-valerobetaine study, with the goal of detecting homocarnitine and acyl-homocarnitines, male and female mice were injected intraperitoneally with 10 mg/kg or 100 mg/kg δ-valerobetaine (DC Chemicals, DC26004) for 7 days as previously described (3). For the mouse tracer study, mice were gavaged with ^13^C_3_-trimethyllysine (Sigma) (25 mg/kg) or trimethyllysine (Sigma) (100 mg/kg) for 3 days as previously described (3). Mice were euthanized, and urine, serum, heart, brain, and liver tissue were collected for liquid chromatography-high-resolution mass spectrometry. For metabolite extraction, 50 μl of mouse serum or urine was combined with 100 μl ice-cold HPLC-grade acetonitrile (Sigma-Aldrich, 900667) containing 2.5% of an isotopically labeled standard mix (23) and then vortexed. Tissues (50 mg) were extracted with 150 μl of ice-cold HPLC-grade acetonitrile: water (80:20) (Sigma-Aldrich, 900682). Samples were incubated on ice for 30 min before centrifugation at 14,000*g* for 10 min at 4 °C. Supernatants were transferred to autosampler vials and placed in an autosampler at 4 °C for mass spectrometry. Mouse S9 liver extracts were prepared at 4 °C by homogenizing liver with 0.1 M potassium phosphate buffer (pH 7.4, including 0.1 mM EDTA, 0.15 M NaCl, protease inhibitor cocktail) before centrifugation at 10,000*g* for 15 min. The supernatant was the S9 fraction.

### Primary cells

Male rat (Sprague-Dawley) cryopreserved plateable hepatocytes (Thermo Fisher, RTCP10) were seeded on collagen-coated plates with Dulbecco’s Modified Eagle’s Medium (DMEM) with GlutaMAX (Gibco, 10564011) containing 10% fetal bovine serum (FBS) and 1% penicillin/streptomycin. After 4 h, the media was changed to William’s E Medium (Gibco, A1217601). Sixteen hours later, media ± 40 μM of etomoxir (Sigma, 236,020), a CPT1a inhibitor, was added. One hour later, the media was changed to include 40 μM palmitate-BSA (Caymen Chemicals, 29,558) ± 100 μM δ-valerobetaine or combined 100 μM δ-valerobetaine and 100 μM meldonium, a BBOX inhibitor (Sigma-Aldrich, M5199). After a 6 h incubation, media was removed and cells were washed twice with ice-cold HBSS and extracted with 200 μl ice-cold HPLC-grade acetonitrile:water (80:20). Extracts were transferred to microcentrifuge tubes for centrifugation at 14,000*g* for 10 min at 4 °C. Supernatants were transferred to autosampler vials for mass spectrometry.

### Cell lines

Human hepatoma Huh7 cell line was transfected with a lentiviral vector containing the sequence for BBOX1 or BBOX1 containing a point mutation by adapting a Huh7 transfection protocol previously developed (62, 63). The Huh7 cells were provided by Dr Arash Grakoui of Emory University and verified by the Emory Integrated Genomics Core facility (63). The lentiviral vector (pLX304-BBOXV5; Clone # HsCD00444260) was obtained from the DNASU plasmid repository center. To control for the potential effects of transfection on homocarnitine generation in the δ-valerobetaine dosing studies, a BBOX^Ala^-Huh7 cell line was developed. The BBOX1 clone was sent to the Emory Integrated Genomics Core facility for a point mutation to induce a change of residue Asp204 to an Ala204. Asp204 is a key residue in the catalytic activity of BBOX as it coordinates with the active site Fe^2+^ (17, 64) (Fig. 2). The mutated cDNA was extracted and incorporated into Stbl3 cells (Invitrogen One Shot Stbl3 Chemically Competent *E. coli*, C737303) and DNA was extracted from the bacteria. The BBOX and BBOX^Ala^ plasmids were transfected with a second-generation lentiviral packaging system consisting of pMD2.G and psPAX2 plasmids into HEK293T cells (Takara Bio, 632180) to generate virus particles. Forty-eight and 72 h after transfection, media containing virus particles were collected and filtered through a 0.45 μm filter, and stored at −80 °C. Huh7 cells were infected with viral media containing 10% FBS and 1% penicillin/streptomycin in DMEM media with polybrene (8 μg/ml) (Sigma-Aldrich, TR-1003) to enhance transduction. Cells were selected with 10 μg/ml blasticidin (InvivoGen, Ant-bl-05) beginning at 24 h after infection and up until the time of the experiment. To confirm the expression of BBOX with the C-terminal V5 tag, the cells were grown to 95 to 100% confluence and the proteins were extracted with cell lysis buffer. The total cell lysates were collected and underwent centrifugation at 12,000*g* for 5 min and then the supernatant was collected and prepared for SDS-PAGE. Immunoblotting was carried out using anti-V5 antibody (1:5000; Sigma, V8137) (63).

### Huh7, BBOX-Huh7, and BBOX-Huh7^Ala^ incubations

All three cell lines were grown in 6-well cell culture plates in DMEM media supplemented with 10% FBS and 1% penicillin/streptomycin until 90% confluency. Media was changed and FBS was reduced to 0.5% 4 h before treatment. Cells were treated with 0 or 100 μM δ-valerobetaine or γ-butyrobetaine (N = 6 each) in culture media for 4 h. In another experiment testing the effects of a δ-valerobetaine and γ-butyrobetaine mixture, BBOX-Huh7 cells were incubated for 4 h (N = 6 for each dose). Incubations were terminated by washing twice with ice-cold HBSS and extracted with 200 μl ice-cold HPLC-grade acetonitrile:water (80:20). Extracts were transferred to microcentrifuge tubes for centrifugation at 14,000*g* for 10 min at 4 °C. Supernatants were transferred to autosampler vials for mass spectrometry. For the dose-response study, HepG2 cells (ATCC HB-8065) were grown identically to the Huh7 cell lines and treated with a range of δ-valerobetaine concentrations (N = 8 each) for 12 h in DMEM media with 0.5% FBS and 1% penicillin/streptomycin. Metabolites were extracted and prepared for mass spectrometry.

For stable isotope tracer experiments in BBOX-Huh7 cells, 272 mg [^13^C_16_]palmitate (Sigma-Aldrich, 605573) was dissolved in 150 mM NaCl and slowly mixed into fatty acid-free bovine serum albumin (BSA) (Sigma-Aldrich, A4602) solution prewarmed to 37 °C. The mixture was heated to 70 °C, and 150 mM NaCl was added with stirring to produce a 1 mM [^13^C_16_]palmitate to 0.17 mM BSA ratio. Huh7 cells were grown in 6-well cell culture plates to 90% confluency. Cell media was replaced with DMEM containing 0.5% FBS and 1% penicillin/streptomycin for 12 h with 200 μM δ-valerobetaine before treatment with [^13^C_16_]palmitate. Cells were washed twice with HBSS, and media was replaced with DMEM media containing 0.5% FBS and 1% penicillin/streptomycin and 200 μM [^13^C_16_] palmitate (N = 5) plus vehicle or 40 μM etomoxir (Sigma, 236,020) (N = 5), a CPT1 inhibitor. After 2 h of incubation, cells were washed twice with ice-cold HBSS and extracted for mass spectrometry.

### Cell-free incubations

The protocol for mouse liver S9 experiments was adapted from previous studies using a 96-well plate format (18). S9 fractions were incubated with 50 μM δ-valerobetaine for 2 h to ensure sufficient levels in this *in vitro* system. After 2 h, half of the wells received vehicle (N = 3) or the BBOX cofactors (16) (N = 3), which included 3 mM 2-oxoglutarate (Sigma-Aldrich, 22202-68-2), 0.25 mM ferrous chloride (Sigma-Aldrich, 372870), and 10 mM sodium ascorbate (Sigma-Aldrich, 134-030-2), and further incubated for 0, 15, 30, or 60 min. Incubations were terminated with ice-cold HPLC-grade acetonitrile, and extracts underwent centrifugation at 14,000*g* for 10 min at 4 °C. Supernatants were transferred to autosampler vials at 4 °C for mass spectrometry.

### Ligand docking and molecular dynamics simulations

BBOX1 bound to γ−butyrobetaine (PDB: 3O2G (17)) was loaded into Maestro software (Schrödinger Release 2024–2). To prepare BBOX for docking and simulations, the protein preparation wizard was used to assign bond orders, add hydrogens, and fill in missing side chains and loops. Default parameters were used for the optimization of hydrogen-bond assignment (sampling of water orientations and use of pH 7.0). Waters beyond 5 Å of heteroatom groups or with fewer than three hydrogen bonds to non-waters were removed. Restrained energy minimization was applied using the OPLS4 force field (65). The receptor grid for docking was generated 10 Å^3^ around γ-butyrobetaine within the BBOX1 active site. Default van der Waals radius scaling parameters were used (scaling factor of 1, partial charge cutoff of 0.25). No other constraints or exclusions were set.

For docking substrates into BBOX1, the structures of γ-butyrobetaine, δ-valerobetaine, (R)-carnitine, and (S)-homocarnitine were drawn using the 2D sketcher in Maestro. The LigPrep wizard was then used to prepare the substrates (by generating possible states at pH 7.0 ± 2.0). The most stringent docking mode (extra precision, “XP”) of Glide (66) was used with flexible docking and post-dock minimization options selected.

Holo-BBOX1 systems for molecular dynamics were built with the system builder panel of Desmond (Schrödinger Release 2024-2). Holo-BBOX1 complexes were neutralized by the addition of Na^+^ counter ions and solvated by a 10 Å orthorhombic box of TIP4P-Ew water (67) with 150 mM NaCl. The force field was set to OPLS4 (65). Solvated systems were loaded into the workspace by using the molecular dynamics panel. The total simulation time for each system was set to 300 ns, with 300 ps trajectory recording intervals, and each system was simulated three times with random seeds for the initial trajectories. The system energy was set to 1.2, and the ensemble class used was NVP. Simulations were set to run at 300.0 K and at 1.01325 bar. The option to relax model systems before simulations was selected. Analysis of the runs was performed with Schrodingers Python API (Schrodinger release 2024-2) as well as in-house Python scripts.

### Mass spectrometry

All mass spectrometry analyses used Thermo Scientific Orbitrap instruments (Velos, Fusion, High Field Q-Exactive, or ID-X) with comparable methods. Minor modifications were made to improve resolution depending on columns, instruments, and purposes. The ESI voltage for positive ionization was set at 3.5 kV with 120,000 resolution and a wide scan range (*m/z* range 85–1275). For MS^2^ ion dissociation, MS^1^ scans were acquired at 60,000 resolution and 30,000 for MS^2^ scans with HCD voltage at 35% NCE for ion dissociation. A Thermo Scientific Ultimate3000 uHPLC was used with the column compartment heated to 60 °C for a Waters Xbridge BEH Amide XP Hydrophilic Interaction Liquid Chromatography (HILIC) column (2.1 mm × 50 mm, 2.6 μm particle size). In other experiments, a Thermo Scientific Vanquish uHPLC was used with a Waters Acquity BEH Amide column (2.1 × 100 mm, 1.7 μm particle size). Mobile phases included HPLC-grade water (A), HPLC-grade acetonitrile (B), and 2% formic acid HPLC-grade water (C). An initial buffer ratio of 22.5% A, 75% B, 2.5% C was held for 1.5-min before ascending to 75% A, 22.5% B, 2.5% C for 4 min and ending with a 1-min gradient hold.

Data from cell experiments and the trimethyllysine tracer mouse study were analyzed using Thermo xCalibur (v.4.2) QualBrowser for peak area determination. All ion dissociation data were analyzed with QualBrowser by averaging scans in the spectra across the chromatographic peak representing the compound of interest. Spectra were analyzed in conjunction with ChemDraw (PerkinElmer v.22.2.0.3348) for theoretical mass calculation of product ion structures. Quantities of homocarnitine, carnitine, and δ-valerobetaine were estimated by reference quantification in Qstd (43). Quality control for cell experiments was assessed with selected metabolites in a quality control standard plasma, Qstd4, pooled from 50 healthy volunteers (Equitech-Bio, SHP45) and analyzed before and after experiments (23). For mouse and human sample analyses, Qstd pooled reference plasma was run before, during, and after 20 sample batches. Data from human samples and the δ-valerobetaine treatment mouse studies underwent mass spectral feature extraction with peak detection, noise removal, alignment, and median summarization of technical triplicates using adaptive processing for LCMS (apLCMS) v.6.3.3 (68) coupled to quality control processing with xMSanalyzer v.2.0.8 (69). False discovery rate (FDR) corrections for an FDR of 5% were performed with Benjamini-Hochberg. Metabolite annotation was performed with xMSannotator (70) using the Human Metabolome Database as reference.

### Structural similarity modeling

A similarity ensemble approach (SEA) was used to examine the structural similarity of δ-valerobetaine to γ-butyrobetaine. SEA works by comparing structures of unknown function to a curated database of proteins and small molecules that are known to interact. Similarity scores are determined by structural resemblance between ligands and then ranked by the likelihood of interaction with known proteins (15).

### Statistical analysis

Group differences for cell and animal experiments were assessed (*p* ≤ 0.05) with an unpaired *t* test or one-way ANOVA and Tukey’s *post hoc* analysis (with GraphPad Prism v.9.5.1).

### Homocarnitine synthesis

Homocarnitine and ^13^C_3_-homocarnitine, with the ^13^C-labels on the trimethylammonio group, were purchased from Chiroblock GmbH (Bitterfeld-Wolfen, Germany) as a custom synthesis project. Structures were verified by mass spectrometry, ^1^H-nuclear magnetic resonance, and chiral chromatography with selection for the L enantiomer, modeled after endogenous L-carnitine.

## Data availability

Raw data not included in this article are available on Metabolomics Workbench. DOIs for these studies are pending. Homocarnitine chemical information: https://pubchem.ncbi.nlm.nih.gov/compound/145650518

## Supporting information

This article contains supporting information (11, 66).

## Conflicts of interest

The authors declare that they have no conflicts of interest with the contents of this paper.

## Supporting information

Supplemental Transition List
