# Supplementary material for: Mammalian hydroxylation of microbiome-derived obesogen, delta-valerobetaine, to homocarnitine, a 5-carbon carnitine analog
Source: J Biol Chem. 2024 Dec 13;301(1):108074. doi: 10.1016/j.jbc.2024.108074 (PMC11773067; doi:10.1016/j.jbc.2024.108074)
Supplement: Supplemental FIgures [file mmc2.pdf]

Supplementary Information

Mammalian hydroxylation of microbiome-derived obesogen, delta-valerobetaine, to homocarnitine, a 5-carbon carnitine analogue

Authors: Jaclyn Weinberg[1], Ken H. Liu[1]*, Choon-Myung Lee[1], William J. Crandall[1], André R. Cuevas[3], Samuel A. Druzak[3], Edward T. Morgan[2], Zachery R. Jarrell[1], Eric A. Ortlund[3], Greg S. Martin[1], Grant Singer[1], Frederick H. Strobel[4], Young-Mi Go[1], Dean P. Jones[1]**

Affiliations:
[1]Division of Pulmonary, Allergy, Critical Care and Sleep Medicine, Department of Medicine, Emory University School of Medicine, Atlanta, GA
[2]Department of Pharmacology and Chemical Biology, Emory University, Atlanta, GA
[3]Department of Biochemistry, Department of Medicine, Emory University, Atlanta, GA
[4] Department of Chemistry, Emory University, Atlanta, GA

*Co-first author; Currently, Department of Chemistry, Emory University, Atlanta, GA
**Corresponding Author

<u>List of contents</u>

Figure S1. In silico prediction of VB-protein interactions.
Figure S2. Interaction times of key BBOX active site residues with substrates and products during molecular simulations.
Figure S3. Western blot confirming ectopic expression of BBOX1-V5 in BBOX-Huh7 and $BBOX^{Ala}$-Huh7 cell line.
Figure S4. Control experiment to confirm activity of BBOX in Huh7, $BBOX^{Ala}$-Huh7, and BBOX-Huh7 cell lines by measurement of carnitine production.
Figure S5. In silico prediction of homocarnitine-protein interactions.
Figure S6. Proposed structures for MS^2 product ions of $^{13}C_{16}$-palmitoyl-homocarnitine, *m/z* 430.4115.
Figure S7. Proposed structures for MS^2 product ions of $^{13}C_2$-acetyl-homocarnitine, *m/z* 220.1454.
Figure S8. Acyl-homocarnitines in primary rat hepatocytes.
Figure S9. Chromatograms and MS^2 spectra of acylated homocarnitines in heart tissue of mice treated with 100 mg/kg δ-valerobetaine.
Figure S10. Proposed structures for MS^2 product ions of acetyl-homocarnitine, *m/z* 218.1387, an isomer of propionyl-carnitine.
Figure S11. Concentration (μM) of homocarnitine in human plasma and urine.
Figure S12. Correlation of *δ*-valerobetaine to homocarnitine in human plasma (N = 223).
Figure S13. Generation of carnitine and homocarnitine when equimolar concentrations of γ-butyrobetaine (BB) and δ-valerobetaine (VB) were added together.
Figure S14. Dose-response δ-valerobetaine cell study.

Supporting Information: Figure S1

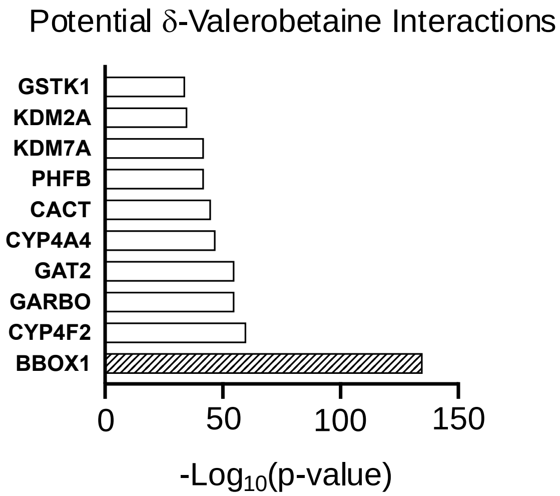

Figure S1. In silico prediction of VB-protein interactions. Similarity ensemble approach (SEA) shows δ-valerobetaine is most likely to interact with the enzyme BBOX1 (highlighted with dashed bars).

Supporting Information: Figure S2

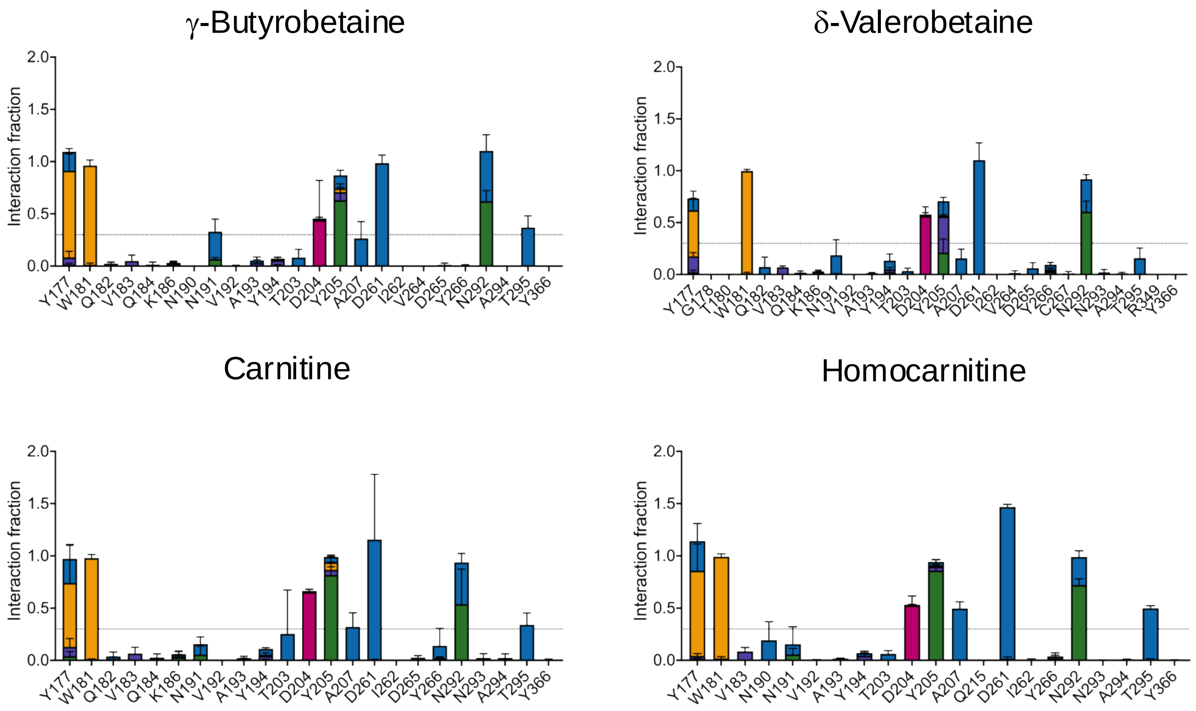

Figure S2. Interaction times of key BBOX active site residues with substrates and products during molecular simulations. BBOX-ligand interactions monitored across three 300 ns molecular simulations. Interaction fraction is the number of frames a contact is made divided by 1000 frames and stacked by subtype. Greater than 100% interaction (1.0 on y-axis) is due to multiple chemical groups on the molecule interacting with the same residue including water networks. Colors indicate interaction subtype. Green = hydrogen bond. Purple = hydrophobic interaction. Pink = ionic interaction. Yellow = cation-pi interaction. Blue = water bridge.

Supporting Information: Figure S3

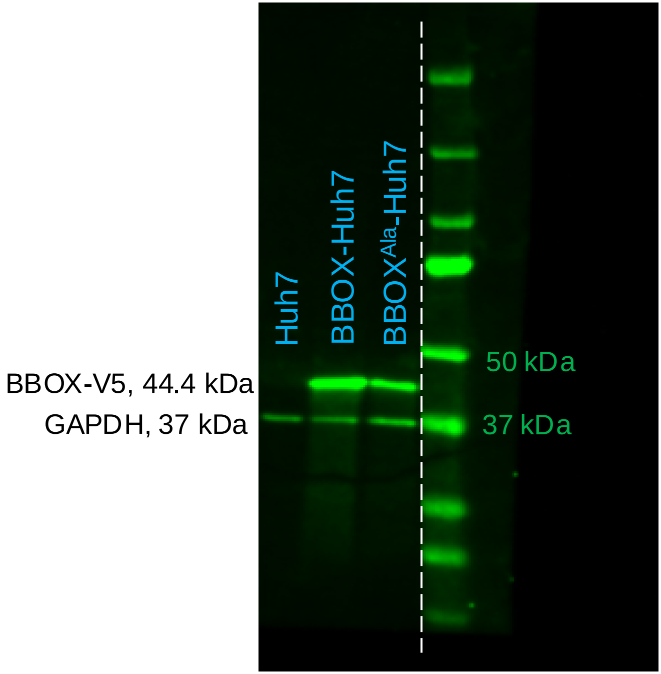

Figure S3. Western blot confirming ectopic expression of BBOX1-V5 in BBOX-Huh7 and BBOX^{Ala}-Huh7 cell line. Cells were generated using lentivirus infection of a V5-tagged BBOX1 constructs into Huh7 cells. Total cell lysate of Huh7 cell lines were separated on SDS-PAGE for immunoblotting. Expression of C-terminal V5 tagged human BBOX1 expression was observed only in transfected cells (44.4 kD). A non-relevant cell line was removed between the BBOX^{Ala}-Huh7 and molecular weight marker lanes and is indicated by the white dashed line.

Supporting Information: Figure S4

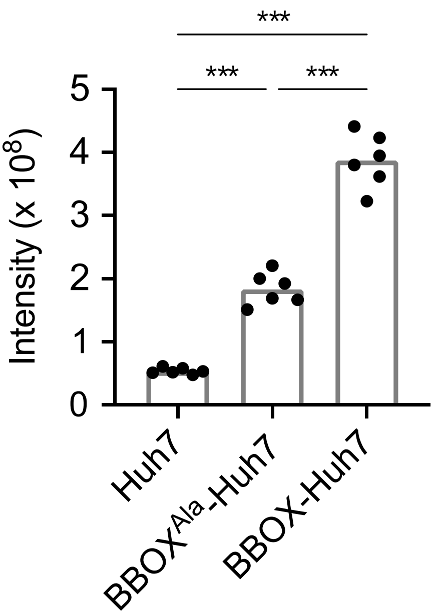

Figure S4. Control experiment to confirm activity of BBOX in Huh7, BBOX^{Ala}-Huh7, and BBOX-Huh7 cell lines by measurement of carnitine production. Cells were incubated for 4 h with 100 μM γ-butyrobetaine and its product, carnitine (*m/z* 162.1125), was measured. $p \leq 0.001$ = **** . Group differences were calculated with a one-way ANOVA followed by Tukey's multiple comparison's test.

Supporting Information: Figure S5

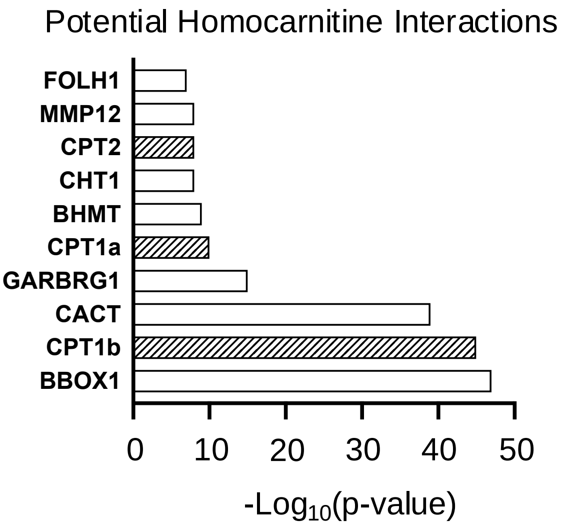

Figure S5. In silico prediction of homocarnitine-protein interactions. Similarity ensemble approach (SEA) shows highlighted enzymes (dashed bars) that are integral to acylation and de-acylation of carnitine in fatty acid oxidation including CPT1a, CPT1b, and CPT2.

Supporting Information: Figure S6

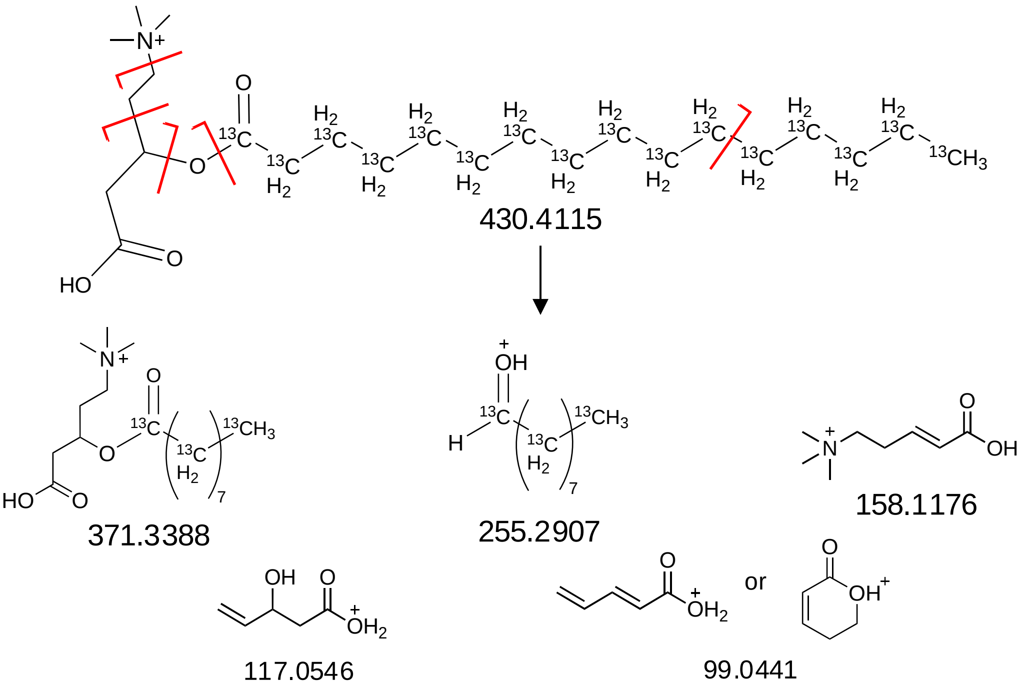

Figure S6. Proposed structures for MS^2 product ions of $^{13}C_{16}$-palmitoyl-homocarnitine, *m/z* 430.4115. Theoretical masses (Da) are listed below each structure. Sites of dissociation are indicated by the red lines.

Supporting Information: Figure S7

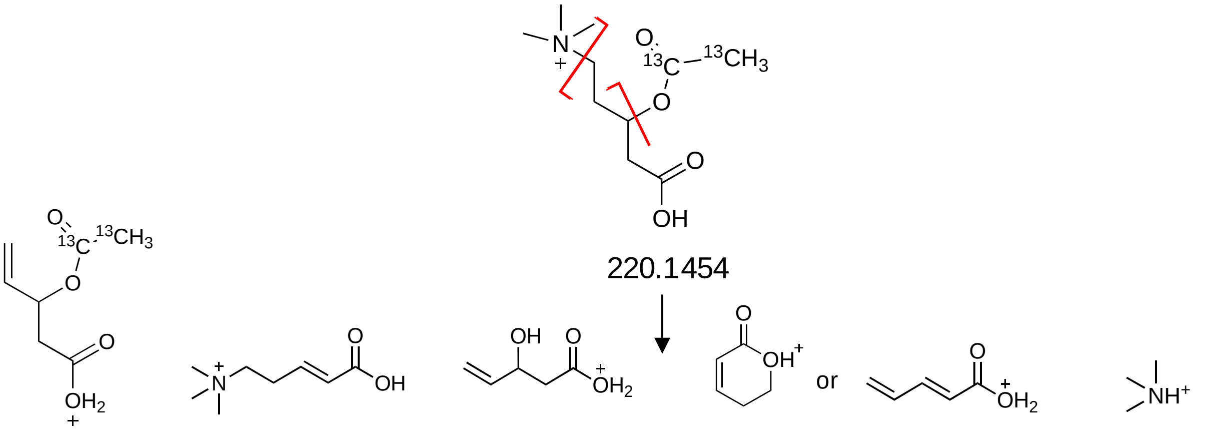

Figure S7. Proposed structures for MS^2 product ions of $^{13}C_2$-acetyl-homocarnitine, *m/z* 220.1454. Theoretical masses (Da) are listed below each structure. Sites of dissociation are indicated by the red lines.

Supporting Information: Figure S8

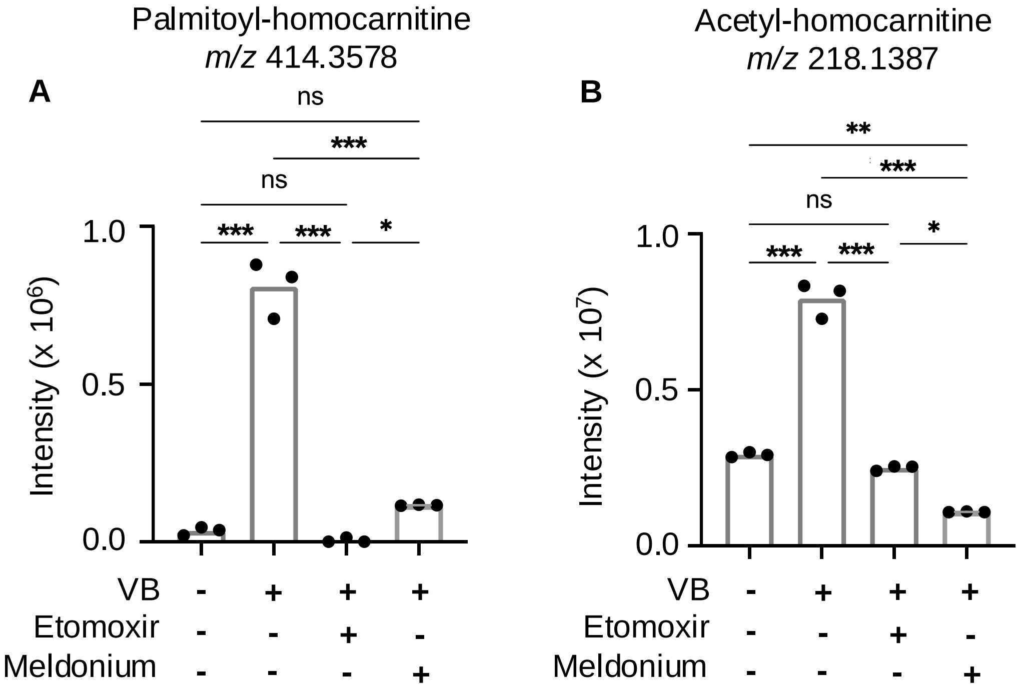

Figure S8. Acyl-homocarnitines in primary rat hepatocytes. (A) In cells incubated with palmitate (40 μM), δ-valerobetaine (VB) treatment (100 μM) increased palmitoyl- and acetyl-homocarnitine production while the addition of carnitine palmitoyl transferase (CPT1a) inhibitor, etomoxir (40 μM), or BBOX inhibitor, meldonium (100 μM), decreased production in a 6 h incubation. In the untreated cells only, the signal of *m/z* 218.1387 was predominantly the isomer, propionyl-carnitine, based on MS^2 ion dissociation. Group differences were calculated with a one-way ANOVA followed by Tukey's multiple comparison's test ($p \leq 0.05$ = *, $p \leq 0.01$ = **, $p \leq 0.001$ = ***, ns = $p > 0.05$).

Supporting Information: Figure S9

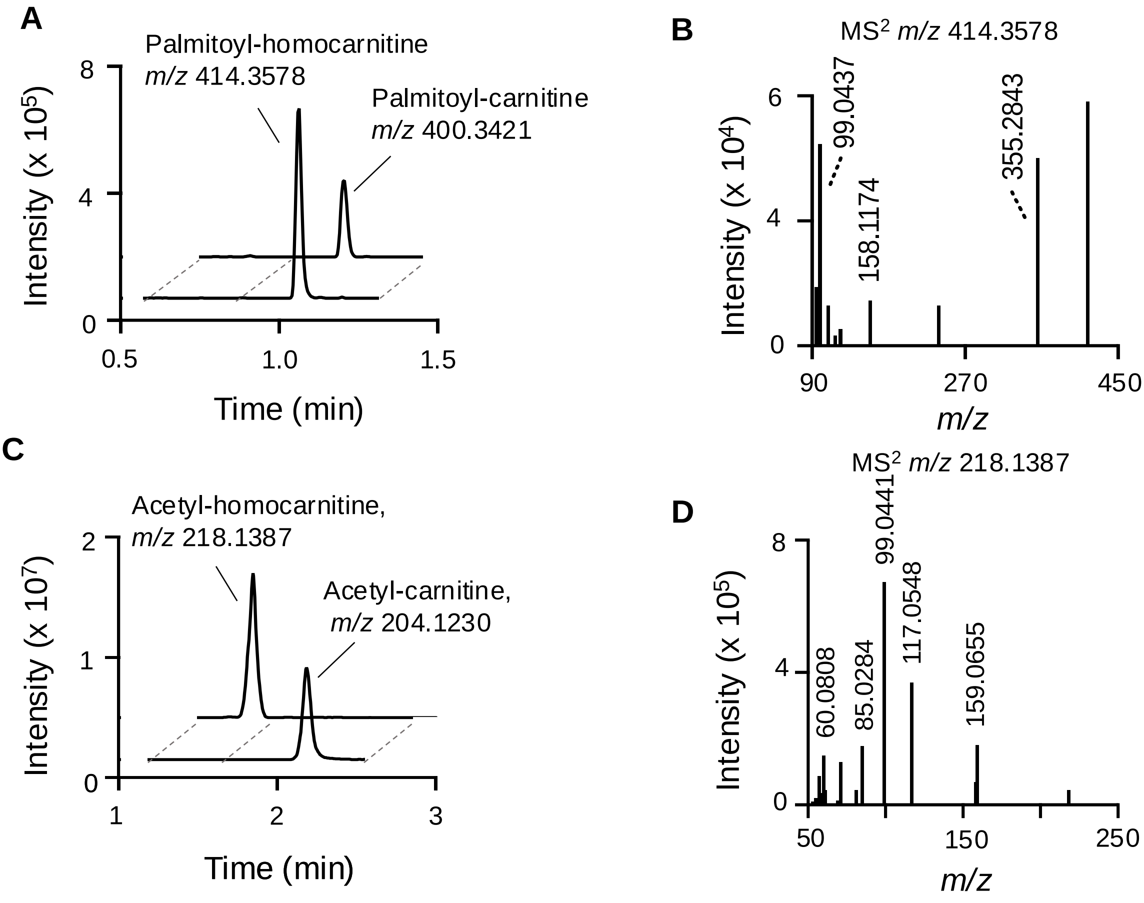

Figure S9. Chromatograms and MS^2 spectra of acylated homocarnitines in heart tissue of mice treated with 100 mg/kg δ-valerobetaine.

Supporting Information: Figure S10

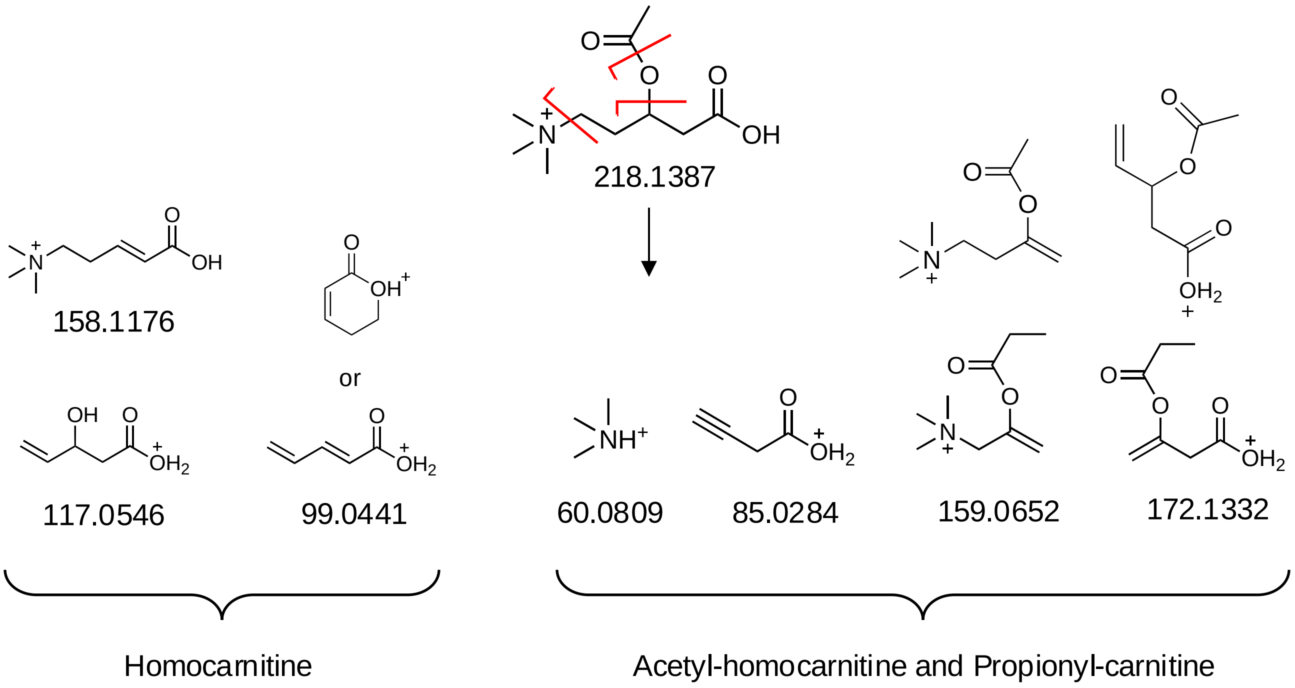

Figure S10. Proposed structures for MS^2 product ions of acetyl-homocarnitine, *m/z* 218.1387*,* an isomer of propionyl-carnitine. Theoretical masses (Da) are listed below each structure. Sites of dissociation for acetyl-homocarnitine are indicated by the red lines. For isomeric acetyl-homocarnitine and propionyl-carnitine product ions, the acetyl-homocarnitine structure is shown on top.

Supporting Information: Figure S11

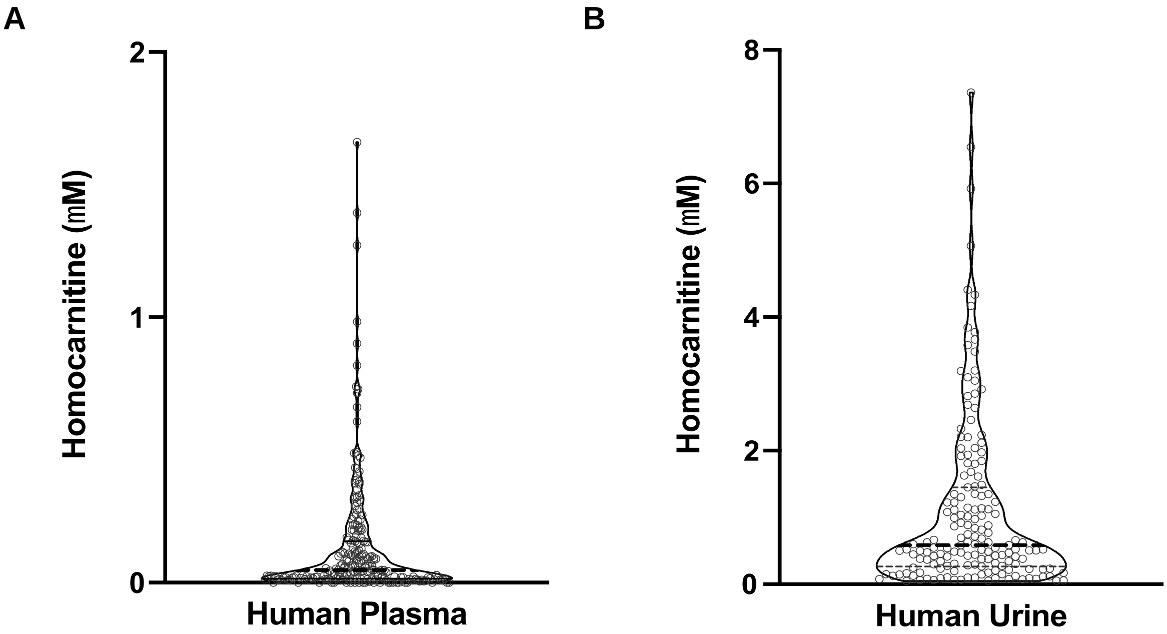

Figure S11. Concentration (μM) of homocarnitine in human plasma and urine. Median (thick black line) and upper and lower quartiles (thin black lines) are shown. In plasma (N = 223), homocarnitine was 0.00 to 1.66 μM with a mean of 0.15 μM and standard deviation of 0.33 μM (A). In urine (N = 165), homocarnitine was 0.05 to 7.36 μM with a mean of 1.11 μM and standard deviation of 1.31 μM (B). Carnitine was used a surrogate standard at the time of analysis. Ionization efficiencies of carnitine and synthetic homocarnitine were equivalent in later tests.

Supporting Information: Figure S12

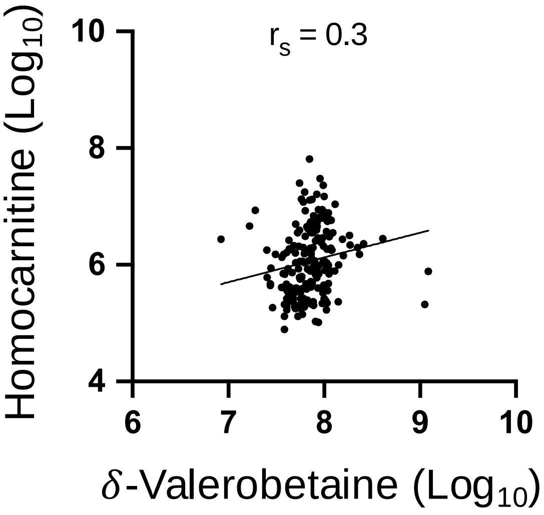

Figure S12. Correlation of δ-valerobetaine to homocarnitine in human plasma (N = 223). Spearman correlation, $r_s = 0.3$, $p < 0.001$.

Supporting Information: Figure S13

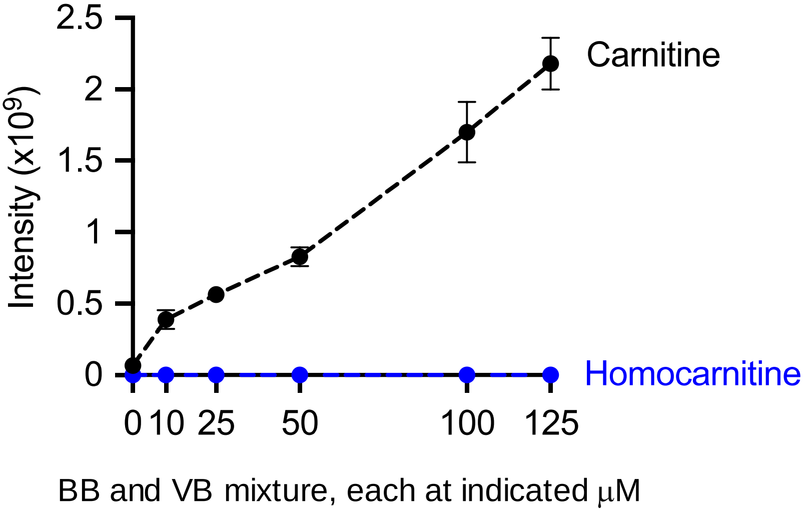

Figure S13. Generation of carnitine and homocarnitine when equimolar concentrations of γ-butyrobetaine (BB) and δ-valerobetaine (VB) were added together. BBOX-Huh7 cells were incubated for 4 h with BB and VB (N = 6 for each dose), and carnitine (*m/z* 162.1125) and homocarnitine (*m/z* 176.1281) were detected Results are consistent with previous BBOX binding studies that showed γ-butyrobetaine has a higher affinity than δ-valerobetaine (2). Homocarnitine generation is likely to occur when δ-valerobetaine levels are higher than γ-butyrobetaine (Fig. S14).

Supporting Information: Figure S14

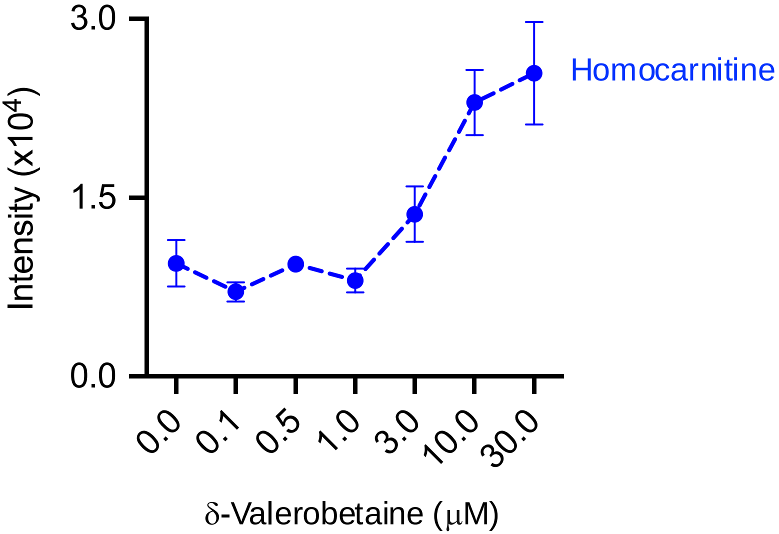

Figure S14. Dose-response δ-valerobetaine cell study. HepG2 cells were incubated for 12 h with δ-valerobetaine (N = 8 for each dose) and homocarnitine (*m/z* 176.1281) was monitored. For comparison, human heart tissue ranges from 3-22 μM (11) δ-valerobetaine and human plasma averages 1 μM δ-valerobetaine (66).
